# Two RR myocardial perfusion acquisition achieves unbiased Myocardial Blood Flow (MBF) estimates

**DOI:** 10.1186/1532-429X-18-S1-W12

**Published:** 2016-01-27

**Authors:** Hui Xue, Michael S Hansen, Sonia Nielles-Vallespin, Andrew E Arai, Peter Kellman

**Affiliations:** grid.94365.3d0000000122975165National Heart, Lung, and Blood Institute, National Institutes of Health, Bethesda, MD USA

## Background

Cardiac perfusion MRI utilizing 2D multi-slice, saturation recovery during Gd first passage is often limited to 3 to 4 slices, especially during stress. A greater number of slices may be acquired by sampling perfusion uptake every two RR intervals, while using a low resolution image acquired every RR for estimating the arterial input function (AIF) [1].This study validates that two RR acquisition can provide sufficient sampling leading to statistically unbiased MBF estimates as the single RR acquisition. With this validation, we demonstrate a high temporal resolution protocol (40 ms imaging duration), capable of acquiring 8 slices in 2RR at heart rates up to 140 bpm with a matrix size of 192 × 112.

## Methods

To validate the hypothesis that two RR sampling is sufficient to capture the myocardial contrast uptake, MBF maps were calculated for stress/rest perfusion studies (N = 16, 8 with FLASH) using our standard imaging protocols: dual-sequence single RR acquisition, saturation recovery, FLASH/SSFP readout, 14°/50° flip angle, FOV 360 × 270 mm^2^, 8 mm slice thickness, 3 SAX, interleaved parallel acceleration R = 3, acquired matrix 192 × 111, ¾ partial Fourier, temporal resolution 53/67 ms. The administrated Gd dose was 0.075 mmol/kg (FLASH) and 0.05 mmol/kg (SSFP). Using a Gadgetron [2] based inline automated workflow, MBF maps were computed for single and 2 RR by discarding alternate heartbeats after a parallel imaging reconstruction. ROIs were drawn in the myocardium and MBF values were compared. In a second experiment, a higher temporal resolution protocol increasing acceleration to R = 4 and 8 SAX slices were prescribed to sample every other RR, leading to 40 ms temporal resolution for FLASH readout. All patient studies were approved by local IRB with written consent. The first imaging experiments were performed on a 3T scanner (MAGNETOM Skyra, Siemens) and the second experiments were performed on a 1.5T scanner (MAGNETOM Area, Siemens).

## Results

**Figure 1:**
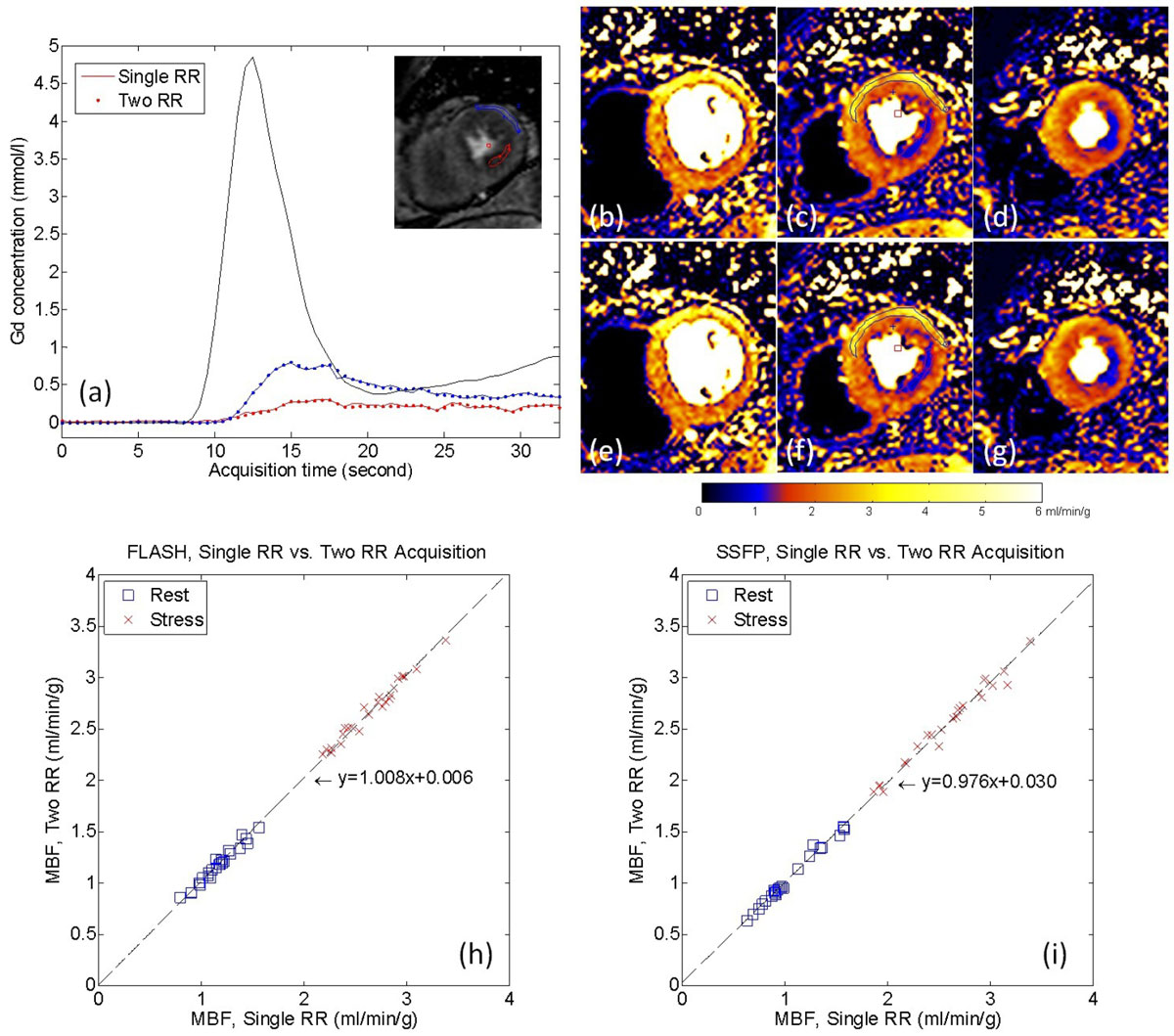
**For a R = 3 stress SSFP perfusion study, the reconstructed perfusion Gd concentration images and time Gd plots after motion correction and surface coil inhomogeneity correction are shown in (a)**. The perfusion signal features are well preserved for myocardium given the two RR sampling. The pixel-wise MBF maps are shown in (b-d) for single RR and (3-g) for Two RR. For two ROIs in the normally perfused and hypoperfused myocardium, the MBFs (ml/min/g) are 3.04 ± 0.56, 0.98 ± 0.17 for singled and 3.03 ± 0.53, 0.98 ± 0.18 for two RR acquisition. For all N = 16 cases, comparison of MBF estimates for single and two RR acquisition are given in (h) and (i). Pixel-wise PMF maps were computed for single and two RR acquisition using a L1 model free deconvolution method. The mean MBF values for all FLASH rest/stress cases are 1.16 ± 0.19/2.65 ± 0.31 (single RR) and 1.17 ± 0;18/2.68 ± 0.29 (two RR). For SSFP, mean MBF values are 10.6 ± 0.30/2.58 ± 0.43 (single RR) and 1.06 ± 0.29/2.56 ± 0.41 (two RR). No significant differences were found (t-test, p-value, FLASH, rest/stress: 0.860/0.711; SSFP, rest/stress: 0.979/0.826).

**Figure 2:**
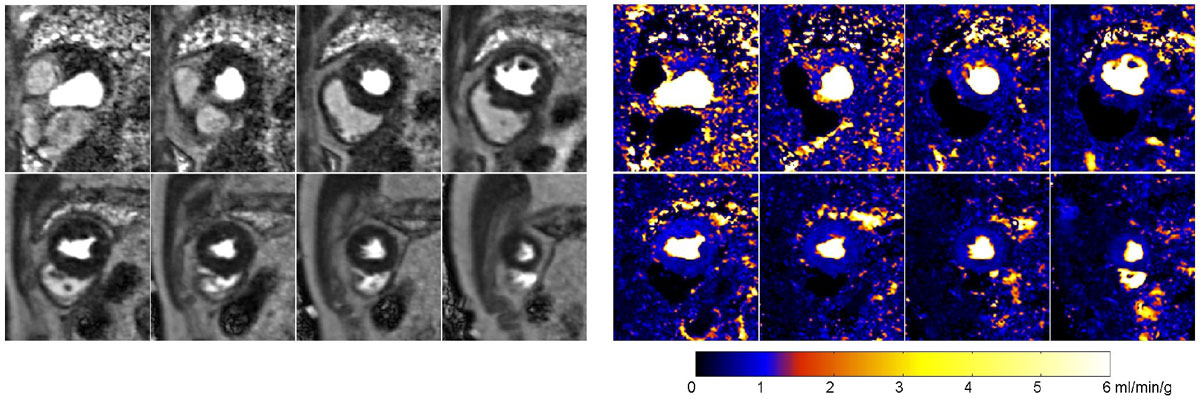
**An example of a 15T rest perfusion study with R = 4, 40 ms temporal resolution, two RR sampling and 8 SAX slices**. The left panel are perfusion Gd images and the right are corresponding pixel-wise MBF maps. With the two RR sampling, high temporal resolution perfusion imaging was achieved with whole myocardium coverage. To improve the image quality of R = 4 acceleration, a motion corrected nonlinear iterative reconstruction algorithm was applied.

An example of R = 3 experiments (Figure 1a-g) compares single RR and two-RR acquisition strategies illustrating that the dynamic characteristics in the time intensity curves are in close agreement. No statistically significant differences (p > 0.5) were found comparing 2RR with single RR maps for both SSFP and FLASH protocols (Figure 1h-i). Figure 2 illustrates that for R = 4 acceleration, nonlinear reconstruction provides sufficient image quality and produced good MBF map. Therefore, combining two RR acquisition with single RR AIF and nonlinear reconstruction, high temporal resolution perfusion imaging is achieved with whole myocardium coverage.

## Conclusions

We validated the hypothesis that two RR sampling with single RR AIF is sufficient to estimate MBF for myocardial perfusion imaging. By combining this strategy with nonlinear reconstruction, 40 ms temporal resolution can be achieved with whole myocardium coverage.
